# Integrating scRNA-seq and bulk RNA-seq to characterize infiltrating cells in the colorectal cancer tumor microenvironment and construct molecular risk models

**DOI:** 10.18632/aging.205263

**Published:** 2023-12-05

**Authors:** Qi Wang, Yi-Fan Zhang, Chen-Long Li, Yang Wang, Li Wu, Xing-Ru Wang, Tai Huang, Ge-Liang Liu, Xing Chen, Qi Yu, Pei-Feng He

**Affiliations:** 1 School of Basic Medical Sciences, Shanxi Medical University, Taiyuan, China; 2 Shanxi Key Laboratory of Big Data for Clinical Decision Research, Taiyuan, China; 3 Key Laboratory of Cellular Physiology at Shanxi Medical University, Ministry of Education, Taiyuan, China; 4 The First clinical Medical College, Shanxi medical University, Taiyuan, China; 5 School of Management, Shanxi Medical University, Taiyuan, China; 6 Department of Anesthesiology, Shanxi Provincial People's Hospital (Fifth Hospital) of Shanxi Medical University, Taiyuan, China; 7 The Fifth Clinical Medical School, Shanxi Medical University, Taiyuan, China; 8 Department of Gastroenterology, The First Hospital of Shanxi Medical University, Taiyuan, China

**Keywords:** colorectal cancer, single-cell RNA sequencing, prognosis, tumour immune microenvironment, chemosensitivity

## Abstract

Colorectal cancer (CRC) is a malignancy that is both highly lethal and heterogeneous. Although the correlation between intra-tumoral genetic and functional heterogeneity and cancer clinical prognosis is well-established, the underlying mechanism in CRC remains inadequately understood. Utilizing scRNA-seq data from GEO database, we re-isolated distinct subsets of cells, constructed a CRC tumor-related cell differentiation trajectory, and conducted cell-cell communication analysis to investigate potential interactions across cell clusters. A prognostic model was built by integrating scRNA-seq results with TCGA bulk RNA-seq data through univariate, LASSO, and multivariate Cox regression analyses. Eleven distinct cell types were identified, with Epithelial cells, Fibroblasts, and Mast cells exhibiting significant differences between CRC and healthy controls. T cells were observed to engage in extensive interactions with other cell types. Utilizing the 741 signature genes, prognostic risk score model was constructed. Patients with high-risk scores exhibited a significant correlation with unfavorable survival outcomes, high-stage tumors, metastasis, and low responsiveness to chemotherapy. The model demonstrated a strong predictive performance across five validation cohorts. Our investigation involved an analysis of the cellular composition and interactions of infiltrates within the microenvironment, and we developed a prognostic model. This model provides valuable insights into the prognosis and therapeutic evaluation of CRC.

## INTRODUCTION

Colorectal cancer (CRC) is a highly lethal and heterogeneous malignancy. The incidence of CRC has been increasing continuously in recent years, contributing a great deal to the global cancer burden. As one of the most common digestive tract tumors, CRC accounted for 9.4% of all cancer-related deaths and is estimated to represent 10% of all cancer diagnoses worldwide [1]. The current main treatment strategy for patients with CRC is surgical resection, combined with perioperative chemotherapy and radiotherapy. Nevertheless, the therapeutic effects are not completely satisfactory, especially when it comes to metastasis and resistance to chemotherapy [2]. With advances in bio-medicine and intensive studies on the pathogenesis of CRC occurrence and progression, several promising emerging therapies are being used in first-line treatments for CRC, including targeted therapy and immunotherapy. The current standard targeted therapy for CRC is anti-vascular endothelial growth factor (anti-VEGF) and anti-epidermal growth factor receptor (anti-EGFR) such as cetuximab [3]. Nivolumab and pembrolizumab, both programmed death-1 (PD-1) inhibitors, are FDA-approved for the treatment of patients with deficient mismatch repair (dMMR) / microsatellite instability-high (MSI-H) metastatic CRC. The clinical trials results showed that the overall response rate (ORR) and disease control rate (DCR) of nivolumab was 55% and 80%, and the ORR and DCR of pembrolizumab were 50% and 89% for dMMR-MSI-H metastatic CRC respectively [4–6]. Nevertheless, there are still many limitations of targeted therapy and immunotherapy in colorectal cancer treatment. The use of anti-EGFR therapies is not beneficial to colorectal cancer patients with mutations in the KRAS, NRAS, or BRAF genes due to resistance that may occur during treatment [7]. PD-1 inhibitors would not benefit the majority of CRC patients, as only 15% of all patients possess MSI-H [8]. It is clear that advances have been made over the last few decades in colorectal cancer treatment, but the long-term survival rate remains unsatisfactory [9].

Over the past few years, the importance of tumor immune microenvironment (TIME) for tumor genesis, treatment resistance, and recurrence has become increasingly apparent. For targeted therapy to be more effective or for treatment strategies to be optimized, enhancing understanding of colorectal cancer heterogeneity and interaction with TIME is crucial to improve patient survival. Our understanding of the molecular events in colorectal cancer has improved considerably during the past decade with the rapid development of next-generation sequencing technologies. Nevertheless, most existing research on RNA is based on bulk RNA sequencing, which involves sequencing a mix of millions of cells at once and obscures individual cell properties. With the advent of single-cell RNA sequencing (scRNA-seq) techniques to analyze the entire mRNA transcriptome at single-cell resolution, we can discern potential cellular heterogeneity and diversity from otherwise homogenous cells [10, 11]. This makes it possible to explore the unique transcriptomic profiles of each cell subpopulation in a large sample and the interactions between cells in the TIME. It is noteworthy that a comprehensive understanding of the TIME characteristics underlying CRC by using scRNA-seq techniques will provide essential insights into the pathogenesis of CRC. Further, it also plays a critical role in the therapeutic response and prognosis of CRC.

In this study, based on scRNA-seq data of CRC from the public database, we re-isolated distinct subsets of cells, constructed the CRC tumor-related cell differentiation trajectory, and investigated potential interactions across cell clusters by using cell-cell communication analysis. Ultimately, we integrate the scRNA-seq data with bulk RNA-seq data from the TCGA database to screen the key genes related to tumor cell subsets in CRC. Furthermore, the prognostic-related genes signature was identified using univariate Cox regression analysis, Lasso regression analysis, and multivariate Cox regression analysis based on the patient clinical information in the TCGA database and validated it in 5 independent GEO cohorts. As a result, we developed a nomogram based on the prognostic-related genes signature and other clinical parameters.

## MATERIALS AND METHODS

### Data collection

**Figure 1:**
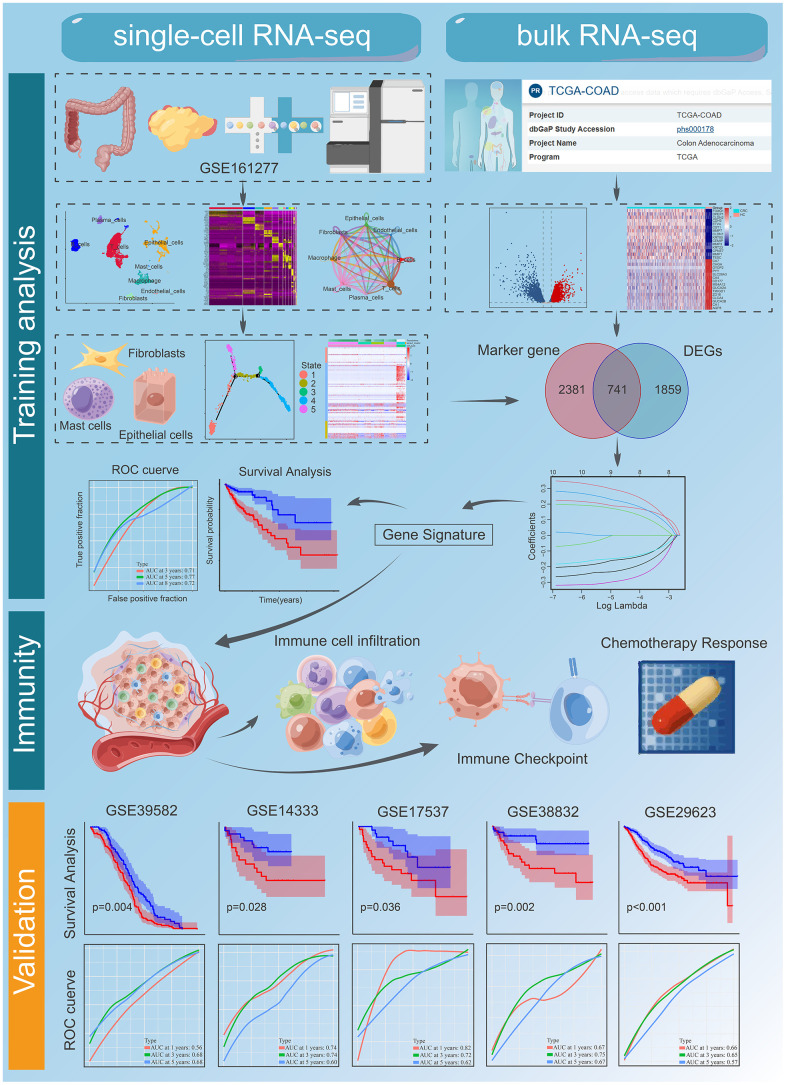
**The workflow of the bioinformatic analysis.** Part of the cartoon graphical was drawn by Figdraw.

Figure 1 summarizes our study workflow. A total of 1558 CRC samples were included in this study, namely, 6 samples with 10×genomics scRNA-seq data were obtained from GSE161277, 521 mRNA sequencing samples and clinical information from the TCGA database, 1,031 CRC microarray samples for five independent cohorts were downloaded from the GEO database, including GSE39582 (n = 563), GSE14333 (n = 226), GSE17537 (n = 55), GSE38832 (n = 122), and GSE29623 (n = 65). More specifically, the 10× genomics scRNA-seq data consists of three normal samples (N1, N2, and N3) and three CRC samples (T1, T2, and T3), which included 3638 cells, 6748 cells, 4050 cells, 3611 cells, 6454 cells, and 2531 cells for each sample. The TCGA RNA-sequencing data was converted into transcripts per log2 (TPM + 1) values, so that they are more comparable with microarrays.

### Single-cell RNA analysis to determine dominant cell types and cell phenotypes

‘Seurat’ package (v4.3.0) [12] was used to merge and analyze the raw gene expression matrix for each sample. Matrices were filtered by retaining cell barcodes with > 1000 UMIs, 500 < expressed genes < 5000 expressed genes or < 10% of reads mapping to mitochondrial RNA. There must be at least three cells expressing each gene, and at least 250 genes expressed in each cell. Normalizing the remaining cells to genes with expression normalized between 0.125 and 3 was performed. By using the ‘FindVariableFeatures’ function, we extract the top 2000 highly variable genes. After regression of confounders, all variable genes were applied in PCA implemented with ‘RunPCA’. Using the ‘FindClusters’ function with dims.use = 1:40 and resolution = 0.1, clusters were identified and visualized with the UMAP [13] dimensional reduction method. Differential gene-expression analysis was performed for each cluster by the Wilcoxon rank sum test using the ‘FindMarkers’ function and setting log2 [Foldchange (FC)] to 0.3 and min.pct to 0.25. The obtained 11 clusters were annotated based on the expression of the marker genes. T cells ((cluster 0, 2,13); CD3D, CD3E, and CD3G), fibroblasts ((cluster 9); COL1A1 and PDGFRB), Mast cells ((cluster 14); TPSAB1 and CPA3), B cells ((cluster 1, 10); CD79A and CD19), plasma cells ((cluster 5); JSRP1), Epithelial cells ((cluster 4, 6, 7, 8); EPCAM), endothelial cells ((cluster 11); EPCAM1), and Macrophages ((cluster 3, 12); CD163, CD68, CD14) (Supplementary Information, Supplementary Figure 1). We conducted Fisher's exact test to identify cell types that were significantly differentially expressed between tumor and normal samples with FC>4 or FC<0.25, p-value <0.05.

### Trajectory inference analysis

Using the ‘Monocle (v2.26.0)’ algorithm, we identified potential differentiation trajectories among differential cell subpopulations [14]. The top 500 combinations of marker genes for each cluster (based on logFC values) were chosen for unsupervised sorting of cells, and the ‘DDRTree’ algorithm was employed for trajectory reconstruction [15]. The BEAM test in Monocle was used to determine which genes have branch-dependent expression dynamics [16].

### Cell-to-cell communication network analysis

In order to uncover the communication interactions between cells and to reveal the mechanism by which molecules communicate, R package ‘CellChat’ [17] was applied. Infer the communication probability at the signaling pathway inferred by calculating the communication probability for all ligand-receptor pairs interactions relevant to each signaling pathway. The ligand–receptor interactions between the epithelial cells, fibroblasts, mast cells, and B cells, Endothelial cells, macrophage, plasma cells, and T cells were mapped using the ‘CellPhoneDB’ R package.

### Functional and pathway enrichment analysis

In order to obtain a deeper understanding of the functional attributes of cell types identified in colorectal cancer, we utilized the ‘ReactomeGSA’ package to perform enrichment analysis on the identified marker genes of eight distinct cell types [18]. Subsequently, we calculated the enrichment scores for each pathway and ranked them based on the difference between the maximum and minimum values of the same pathway across the eight cell types. Ultimately, we conducted further analysis on the top 20 pathways exhibiting the greatest differences. To further characterize the pathway and functional differences between the high and low-risk groups identified by the prognostic signature, we employed the 'ClusterProfiler' package for conducting GO and KEGG enrichment analysis, which is based on all differentially expressed genes [19].

### Prognostic signature construction and validation

Transcriptomic data from the CRC cohort were used for joining with single cell data to construct risk prognostic models. Gene expression differences between tumor tissue and adjacent normal tissue are identified using the ‘limma’ package. The intersection of DEGs and marker genes of differential cell subsets to construct the prognosis risk model. A univariate Cox regression analysis was performed to identify prognostic value genes with p-value < 0.01. Next, we applied the LASSO regression analysis to select key genes [20] using ‘glmnet’ R package. Finally, a multivariate Cox proportional regression model was used to construct a prognostic model. A risk score can be calculated as follows: Risk score = (expression of gene_1_ × β_1_) + (expression of gene_2_ × β_2_) + … (expression of gene_n_ × β_n_). The “β” represents the coefficient of selected genes and “expression” represents their expression level. The prognostic model was validated by five independent cohorts of external validation from GEO. Risk scores were normalized using the Z-score method in the training and validation cohort. Using a median risk score, patients were classified into low-risk and high-risk groups. We visualized the DEGs using the R package ‘ggplot2’. The Kaplan-Meier survival curve (KM) was used to assess the difference in survival between the two risk groups using the ‘survminer’ R package. Receiver operating characteristic curves (ROC curves) were used to evaluate model performance.

### Analysis of clinical features and independent prognostic

To detect differences in clinical features between the two risk groups, Wilcox. tests and Kruskal-Walli’s analysis were performed. Clinical features including age, gender, T stage, N stage, M stage, and clinical stage. To determine the prognostic value of risk scores and clinicopathological factors, we performed univariate and multivariate Cox regression analyses.

### Immune cells infiltration and immune function analysis

To characterize the TME in different risk groups, we made a comparison of the expression levels of immune cells and immune-related pathways. The activity of immune-related pathways from ‘MSigDB C2 gene set’ (c2.cp.kegg.v7.0.symbols.gmt) was used as a reference for ssGSEA, performed using implementation by ‘GSVA’ and ‘GSEABase’ R package. The ‘CIBERSORT’ was used to estimate the immune cell expression, based on 22 types of flow-purified immune cells [21].

### Drug susceptibility analysis

To provide insight into the impact of risk scores on drug sensitivity in prognostic model, the R package ‘pRRophetic’ was employed to evaluate response to chemotherapeutic agents based on the 50% maximum inhibitory concentration (IC50) using data from the Genomics of Drug Sensitivity in Cancer (GDSC) database.

### Immunohistochemistry and protein level validation

To further validate the protein expression levels of prognostic signatures in normal tissues and colorectal cancer, immunohistochemistry (IHC) staining images were downloaded from the Human Protein Atlas [22] (HPA version 22.0, http://www.proteinatlas.org) which included proteome analysis based on 27397 antibodies targeting and 17291 unique proteins.

### Statistical analysis

We used R software version 4.2.0 to analyze and generate figures. P-value < 0.05 was considered significant, unless otherwise noted.

### Data availability statement

The datasets supporting the conclusions of this article are available in the GEO repository, [https://www.ncbi.nlm.nih.gov/geo/]. The names of the repository/repositories and accession number(s) can be found in the article.

## RESULTS

### Single-cell atlas of CRC

**Figure 2:**
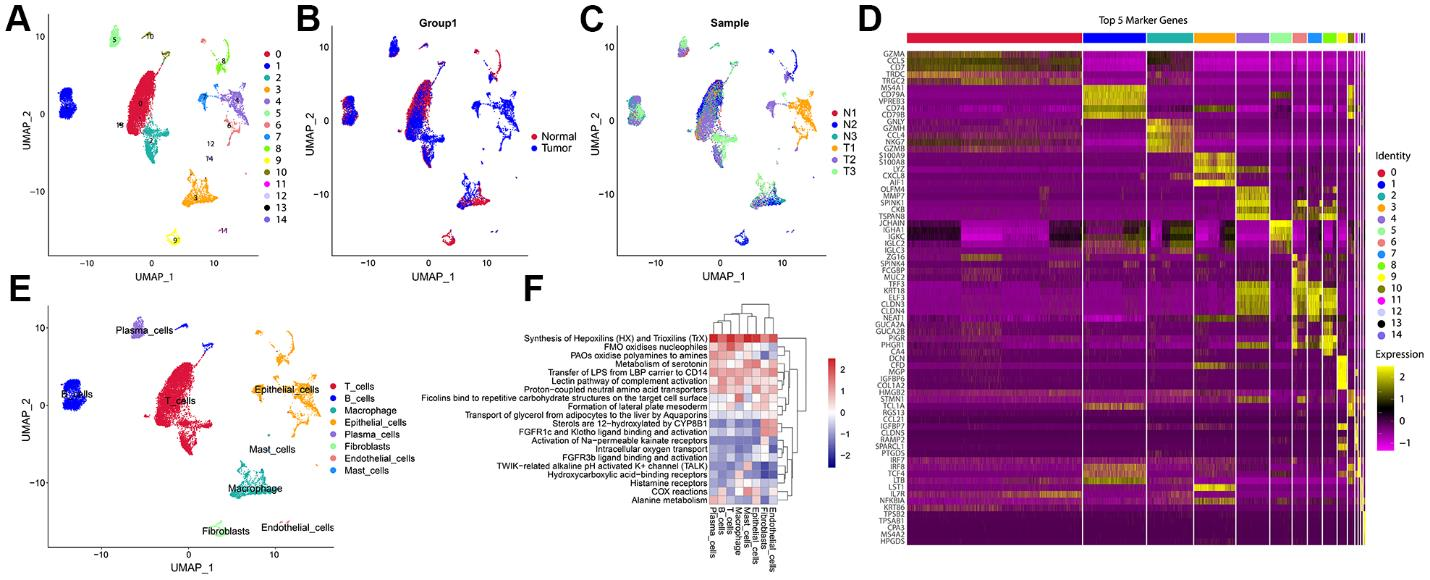
**A Single-cell atlas of CRC.** (**A**) UMAP representation of scRNA-seq from CRC cells reveals 15 distinct clusters. (**B**) UMAP dimensional reduction visualizations single cells from tumour tissues and normal control tissues. (**C**) UMAP dimensional reduction visualizations single cells from six types of samples. (**D**) The heatmap showed the relative expression of genes in 15 clusters. The color yellow represents genes that are highly expressed, and the color purple represents genes that are lowly expressed. (**E**) Eight major cell types identified in CRC. (**F**) Pathway gene set enrichment analysis of the expression profiles for each cell-type.

We obtained 27,034 single-cell gene expression profiles from 6 samples (CRC samples (n = 3) and normal samples (n = 3)). We retained 16,626 single cells for downstream analysis after quality control (Supplementary Figure 2A, 2B). Principal component analysis (PCA) was performed after normalization to assign all cells to different clusters based on their expression of 2000 variable genes (Supplementary Figure 2C, 2D). Finally, 15 clusters were found (Figure 2A). Figure 2B shows a summary of single cells from six types of samples. Cells originating from tumour tissues and normal control tissues are shown in Figure 2C. In addition, significant expression marker genes were identified using logFC > 0.3 and adj Pval < 0.05 as thresholds, and the top 5 significant differential markers for each cluster were shown by heatmap (Figure 2D). According to the Uniform Manifold Approximation and Projection (UMAP) analysis and canonical markers expression, eight distinct cell populations were identified (Figures 2E and Supplementary Figure 1), including the T cells, B cells, Macrophage, epithelial cells, Plasma cells, Fibroblasts, Endothelial cells, and Mast cells. A total of 9569 differential expression genes between these clusters (Supplementary Table 1). The above results reveal the heterogeneity of CRC tumor microenvironment.

### Functional enrichment analysis of cell types identified in CRC

‘ReactomeGSA’ functional enrichment revealed that the eight cell types are predominantly positively enriched biological processes including Synthesis of Hepoxilins (HX) and Trioxilins (TrX), Metabolism of serotonin, Transfer of LPS from LBP carrier to CD14, Proton-coupled neutral amino acid transporters. Epithelial cells, Mast cells, Macrophage, T cells, B cells, and Plasma cells were negatively enriched in Sterols are 12-hydroxylated by CYP8B1, FGFR1c and Klotho ligand binding and activation, and Activation of Na-permeable kainate receptors (Figure 2F).

### CRC have different patterns of cell-cell communication

**Figure 3:**
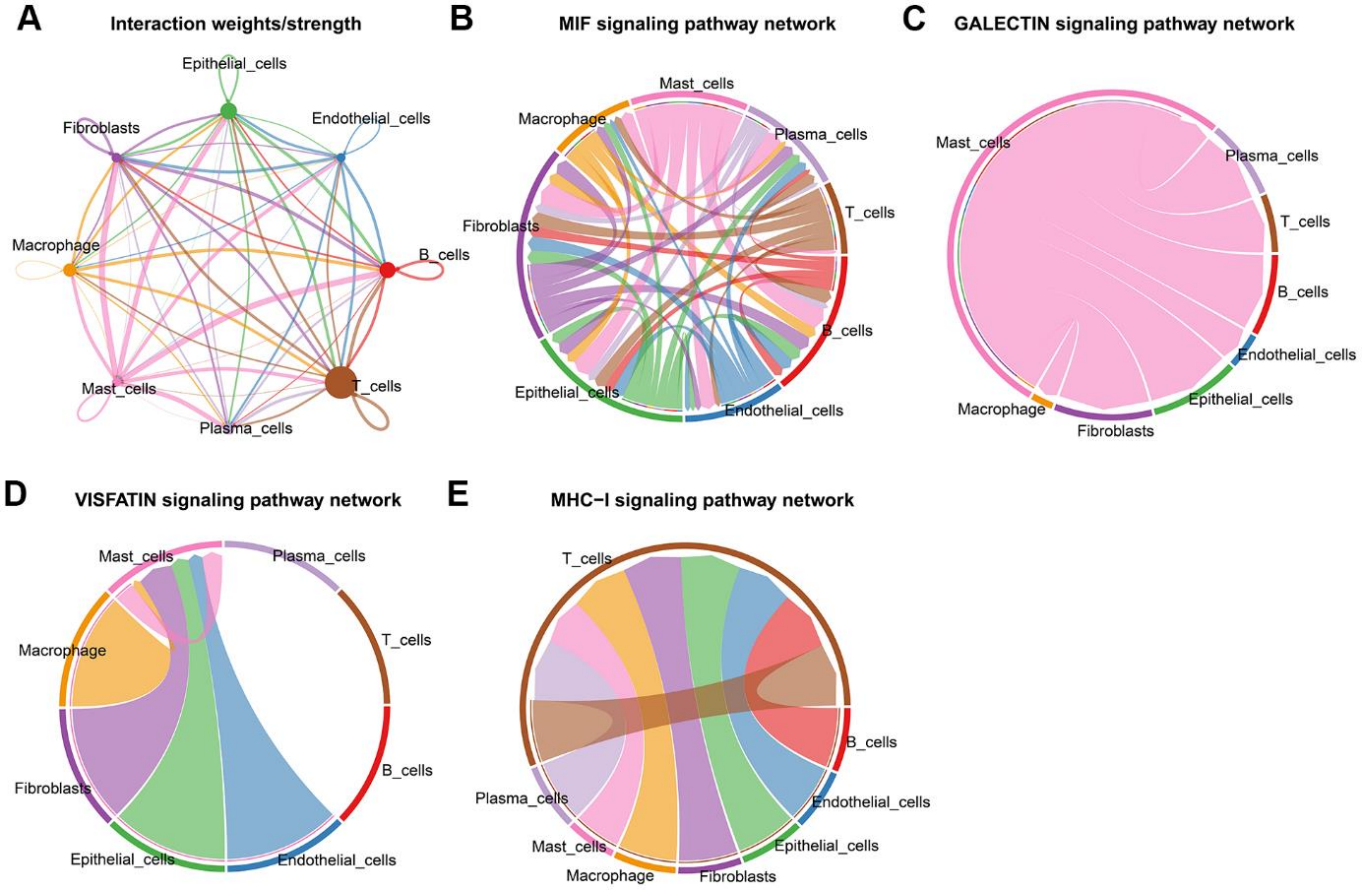
**Cell-cell communications in CRC.** (**A**) Integrated cell-cell communications network plotted by interaction weighted. (**B**–**E**) Cell-cell interaction signaling pathways.

Recent studies have emphasized the importance of intercellular communication in the progression of various tumors. We have carried out a cell-cell communication analysis between the cell subgroups. This approach maps the expression of ligand-receptor pairs between different immune cells in TME and allows inference of potential cell-cell interactions. Of them, we noted several trends in T cells showed strong communication with B cells, Endothelial cells, Epithelial cells, Fibroblasts, Macrophage, Mast cells, and Plasma cells (Figure 3A). Macrophage, Fibroblasts, and Mast cells were second. These pathways, including MIF signaling pathway, GALECTIN signaling pathway, VISFATIN signaling pathway, and MHC−I signaling pathway, might play a role in the cell-cell communication (Figure 3B–3E). CD74-CXCR4 has crucial roles in the MIF signaling pathway. NAMPT_INSR, LGALS9_CD45, and HLA-A_CD8A play crucial roles in the VISFATIN signaling pathway, GALECTIN signaling pathway, MHC−I signaling pathway, respectively (Supplementary Figure 3). The results of this study demonstrate that CRC's unique TME may be shaped by TME-specific cellular communication.

### CRC have different patterns of cell trajectory

**Figure 4:**
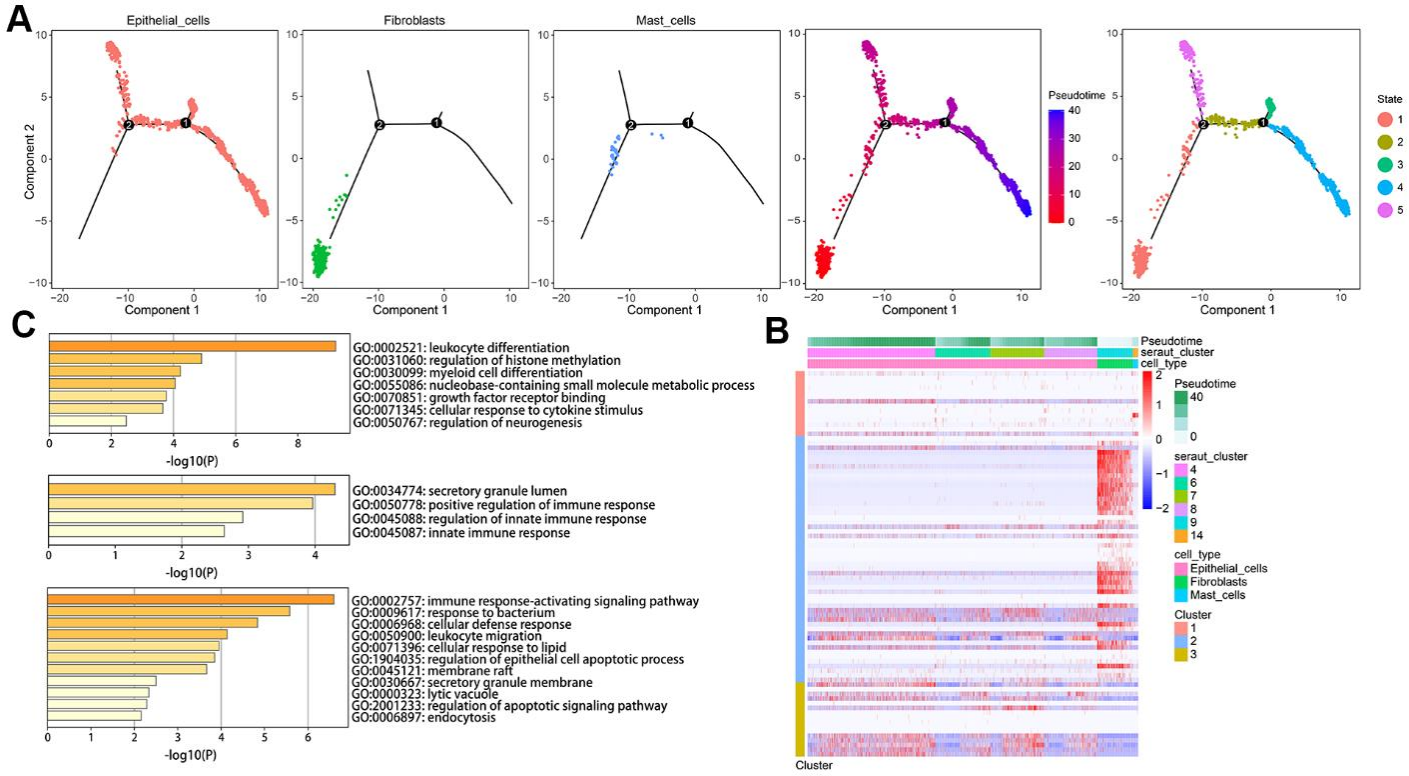
**Developmental trajectory of cells in CRC.** (**A**) Cell trajectory and pseudo-time analysis for the identified hub cell types. (**B**) Heatmap of gene expression profiles according to pseudotime trajectory. (**C**) The top annotated GO biological processes terms in each cluster were provided.

We identified three significant cell types and performed pseudotime analysis, which implied that fibroblasts were located at the beginning of cellular evolution on this map. The trajectory of the Epithelial cells yields five levels of development (states 1-5). Mast cells were located at the state 1 and state 2 (Figure 4A). Our next step was to calculate the contribution of genes to cell development, and the top 100 genes were selected for visualization (Figure 4B). Accompanying the cellular trajectory of three cells, GO enrichment analysis revealed that the processes including response to regulation of innate immune response and immune response-activating signaling pathway were increased, whereas leukocyte differentiation and growth factor receptor binding were reduced (Figure 4C).

### RNA-Seq data analysis to identify differentially expressed genes

**Figure 5:**
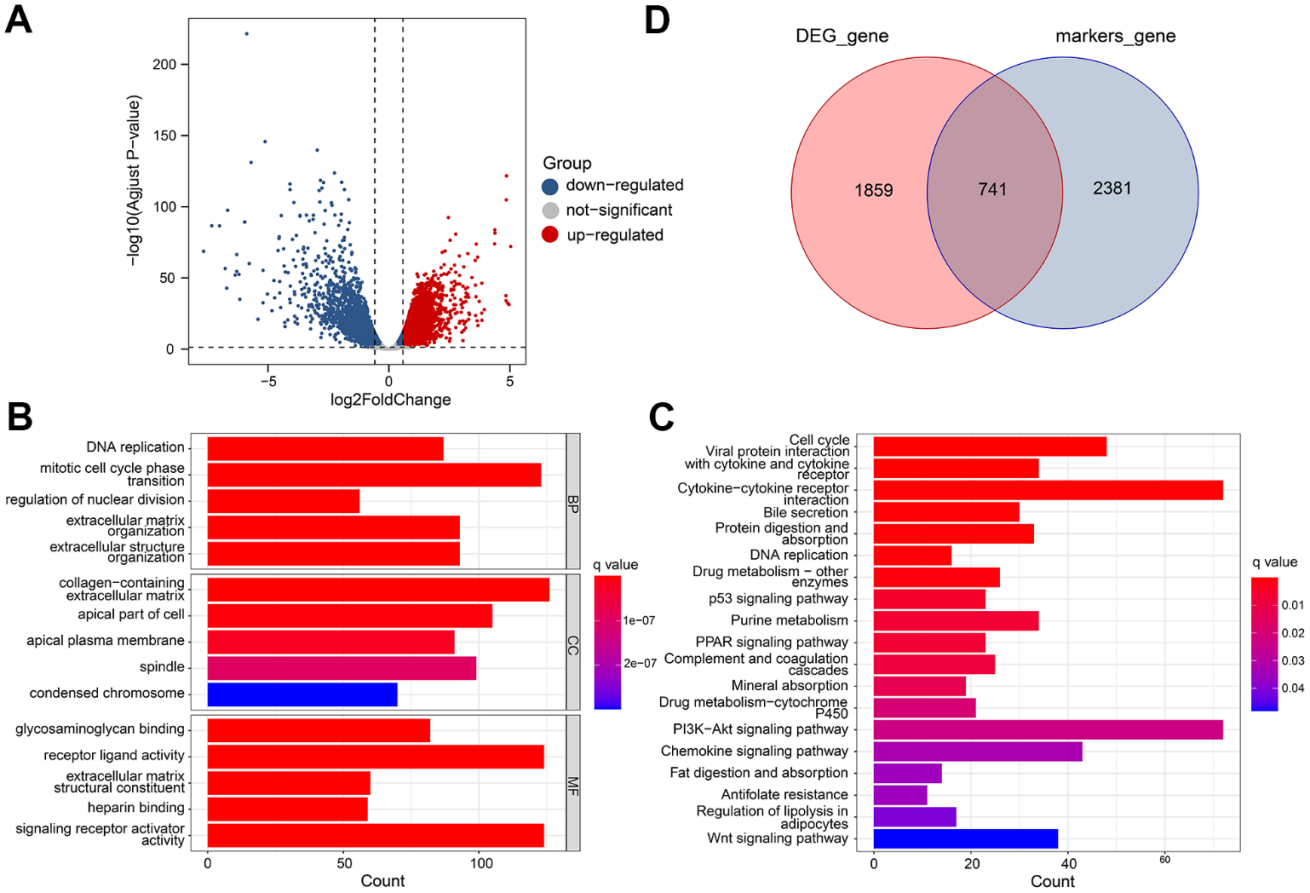
**Differential gene analysis of in TCGA datasets.** (**A**) A volcano plot showing the DEGs in the TCGA cohort that are up-regulated and down-regulated. (**B**, **C**) GO and KEGG enrichment analysis of the identified DEGs. (**D**) Venn diagram analysis of DEGs and marker genes.

In TCGA datasets, we identified 2600 differential expression genes (DEGs) between adjacent normal and tumor tissues (Figure 5A). There were 1455 genes that were up-regulated, and 1145 that were downregulated. Based on GO analysis, the DEGs were enriched primarily in biological processes of DNA replication, collagen-containing extracellular matrix, and glycosaminoglycan binging (Figure 5B). Analysis of KEGG data revealed that DEGs were predominantly enriched for Vital protein interaction with cytokine and cytokine receptor and Bile secretion (Figure 5C). Finally, 741 gene expression matrices were obtained by taking the intersection using marker genes and DEGs (Figure 5D).

### Prognostic model construction and validation

**Figure 6:**
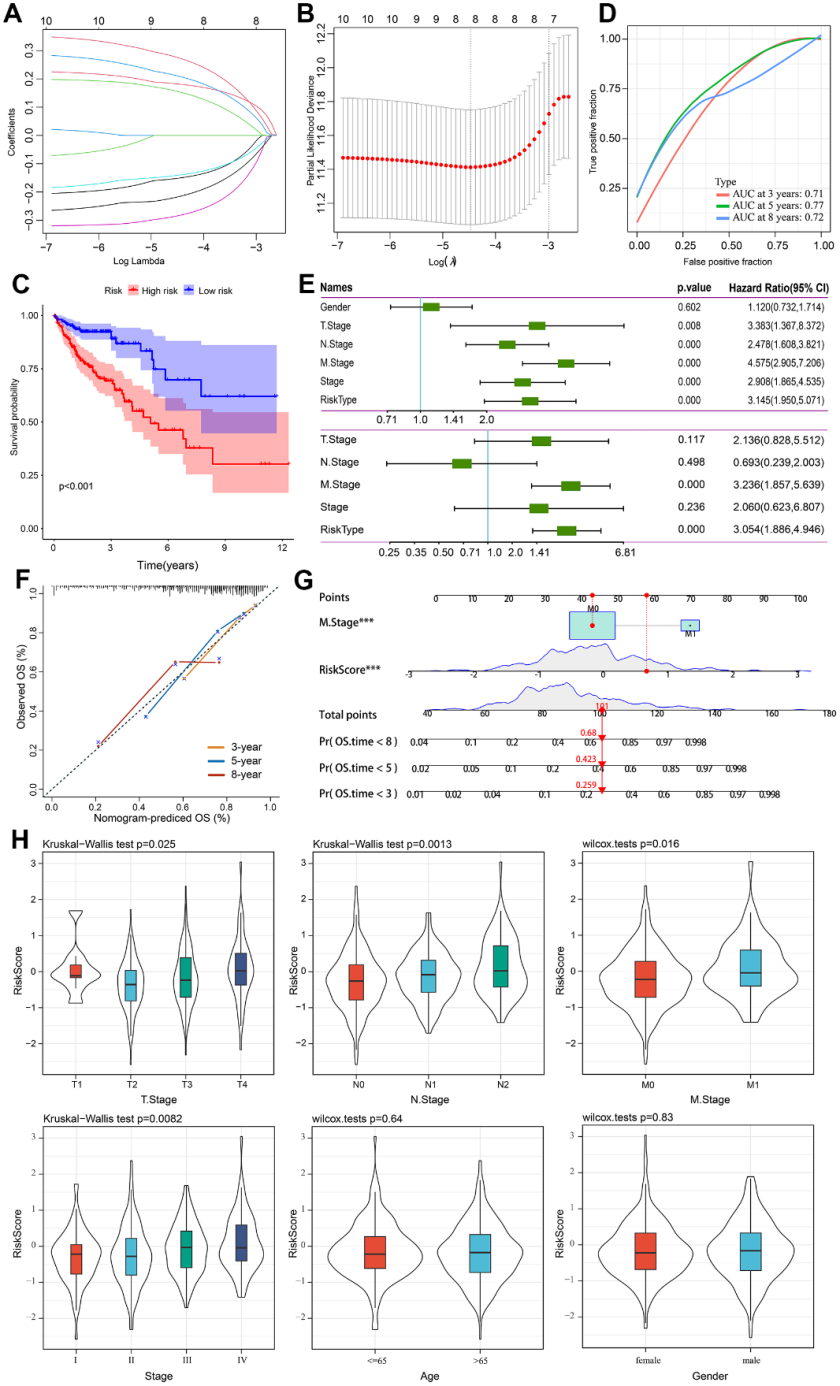
**Prognostic model establishment and validation for patients with CRC.** (**A**) Cross-validation for eight OS-related genes in the LASSO regression. (**B**) Partial likelihood deviance for the LASSO regression for eight candidate genes. (**C**) The Kaplan-Meier curve was used to analyze the OS of two risk groups of patients with CRC. (**D**) ROC curves evaluate the predictive ability of the constructed risk model. (**E**) The results of univariate independent prognostic analysis and multivariate independent prognostic analysis. (**F**) The nomogram’s calibration curves for predicting 3-, 5-, and 8-year OS in TCGA-CRC cohorts. (**G**) Based on independent prognostic factors, a nomogram was developed to predict overall survival. The survival rate at 3-, 5-, and 8-year survival rate is predicted according to the total score. (**H**) The relationship between risk score and common clinicopathological characteristics.

Using 741 gene expression profiles, we conducted univariate Cox regression analysis to identify potential prognostic genes. Ten genes were identified as prognostic. Through LASSO regression analysis, eight genes were identified in the risk model (Figure 6A, 6B). An eight-genes prognostic signature was constructed using Multivariate Cox regression analysis, including ETS2, SEZ6L2, TRIP6, ATOH1, CXXC5, CLDN23, PCOLCE2, and DPP7. Our risk score was calculated using the following formula in TCGA cohort based on their coefficients. risk score = expression level of ETS2 * (-0.172) + expression level of SEZ6L2* 0.332 + expression level of TRIP6* 0.203 + expression level of ATOH1* (−0.163) + expression level of CXXC5*(−0.319) + expression level of CLDN23 * (-0.261) + expression level of PCOLCE2*0.201 + expression level of DPP7 *0.258. Patients were divided into high- and low-risk groups according to their median risk scores. The survival curve showed that high-risk patients had a shorter OS than low-risk ones (Figure 6C). The time-dependent ROC curve analysis demonstrated that the model has precise predictive ability, with areas under curves (AUCs) of 0.71, 0.77, and 0.72 for 3, 5, and 8 years, respectively (Figure 6D). A single-factor and multi-factor Cox analysis was performed to determine whether the risk score could predict the outcome of CRC patients. Risk scores could be used as independent prognostic factors (Figure 6E). As a result, we developed a nomogram based on other independent prognostic factors to predict overall survival of CRC patients (Figure 6G), which was subsequently validated using calibration plots (Figure 6F). Afterwards, we examined the clinicopathological characteristics of the patient and the risk score. There are substantial differences in T stage, N stage, M stage, and stage between the two groups (Figure 6H).

### External validation of the robustness of the risk model

**Figure 7:**
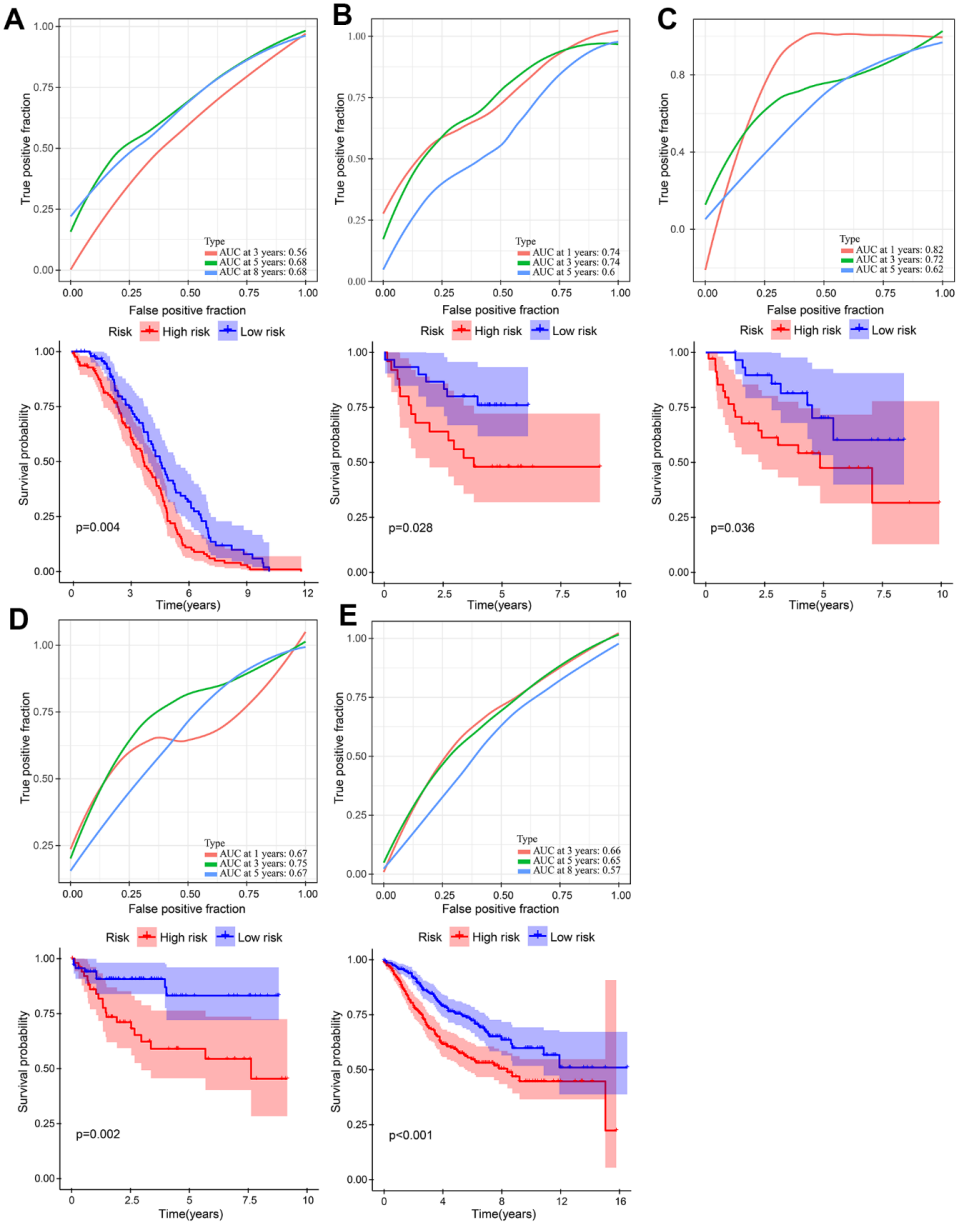
**Validation of the gene signature in five independent GEO cohorts.** (**A**) GSE14333 (n = 226). (**B**) GSE17537 (n = 55). (**C**) GSE29623 (n = 65). (**D**) GSE38832 (n = 122). (**E**) GSE39582 (n = 563).

Five independent GEO cohorts were included in this study to validate the robustness of the risk model. Using the same method, risk scores were calculated for each patient in five GEO cohorts. According to Kaplan-Meier analysis, each of the five cohorts had a worse prognosis than each of the low-risk groups. namely, GSE14333 (Figure 7A, P = 0.004), GSE17537 (Figure 7B, P = 0.028), GSE29623 (Figure 7C, P = 0.036), GSE38832 (Figure 7D, P = 0.002), and GSE39582 (Figure 7E, P < 0.001). A good performance was also shown by the ROC curves of the risk model. There is a maximum area under ROC curve of 0.82. There can be more than 0.7 area under the ROC curve. It is evident that the model is well-regularized by the validation performance, which is consistent with the training performance.

### The immune function between high- and low-risk groups

**Figure 8:**
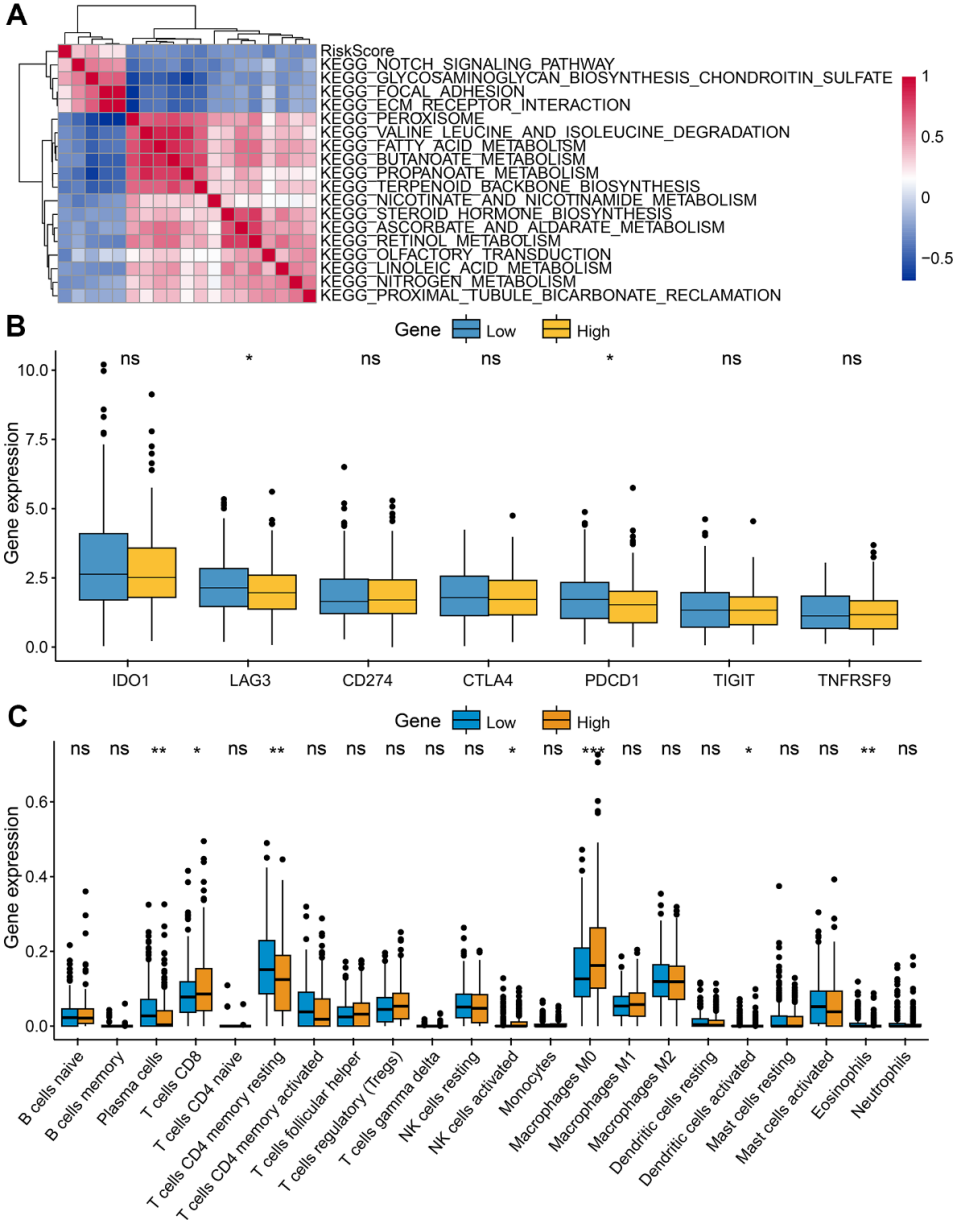
**Characteristics of tumor immune microenvironment.** (**A**) Heatmap showed the relationship between risk score and immune functions. (**B**) Immune checkpoint expressed differently between low-risk and high-risk groups. (**C**) Tumor-infiltrating immune cells expressed differently between low-risk and high-risk groups.

Further research was conducted on the relationship between two risk groups and TME. We identify pathways that were significantly enriched in low- and high- risk groups using GSEA. Genes in the high-risk group were significantly enriched in notch signaling pathways, glycosaminoglycan biosynthesis chondroitin sulfate, and ecm receptor interaction. However, genes in the low-risk group were significantly enriched in terpenoid backbone biosynthesis, olfactory transduction, and peroxisome (Figure 8A). The expression of LAG3 and PDCD1 was higher in the low-risk group (Figure 8B), suggesting that this group was more responsive to immune checkpoint inhibitors. For the exploration of CRC's immune landscape, we chose 22 different types of immune cells. The percentage of T cells CD8, Macrophages M0, Plasma cells, NK cells activated, dendritic cells activated, and eosinophils in both groups differed significantly (p < 0.05) (Figure 8C).

### Drug sensitivity analysis

**Figure 9:**
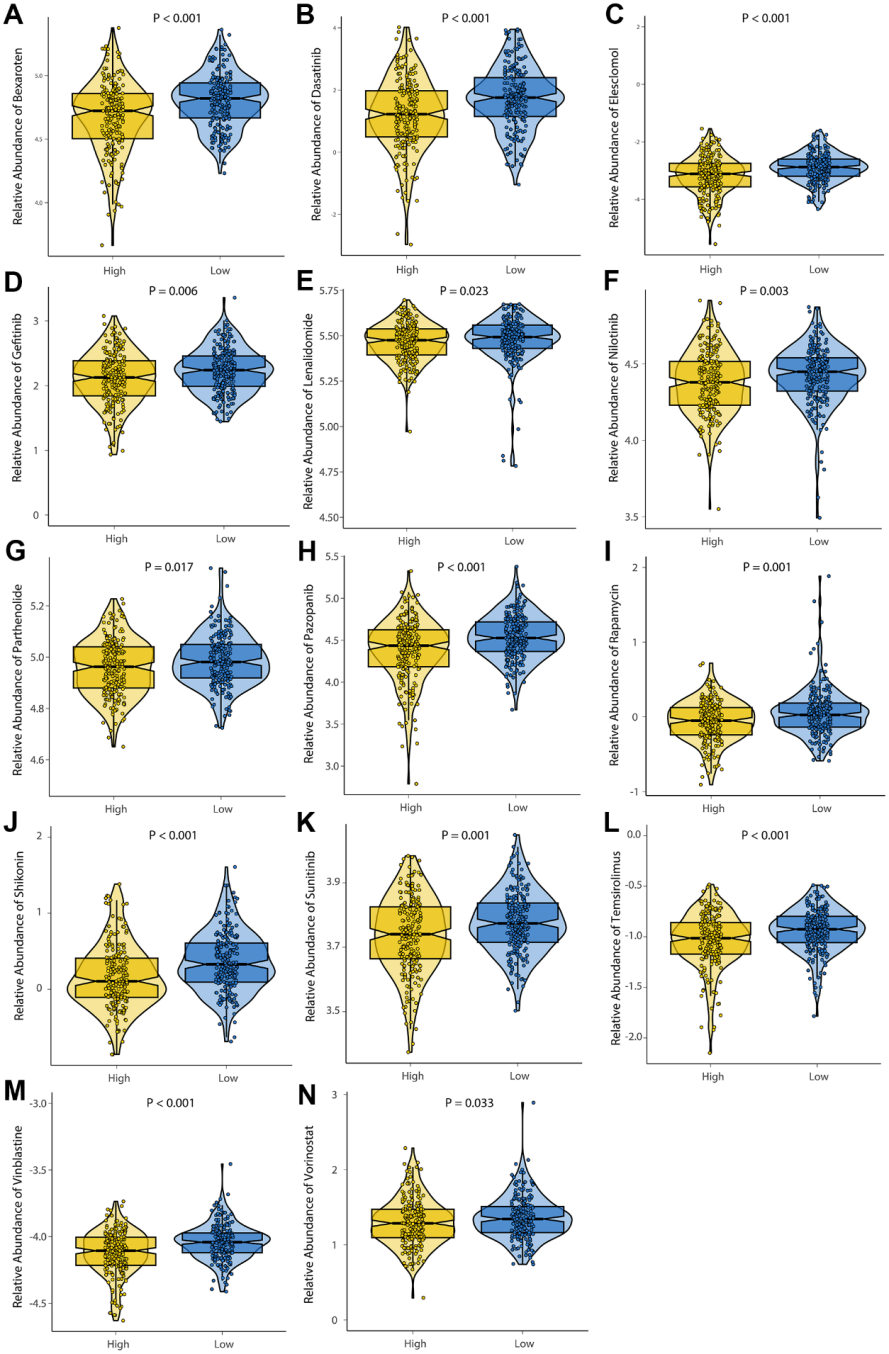
**Evaluation of drug sensitivity.** The comparisons in IC50 value between low-risk and high-risk groups. The ordinate shows the IC50 value of anticancer drug target sensitivity. Lower IC50 values are associated with higher sensitivity to the anticancer drug. (**A**) Bexaroten. (**B**) Dasatinib. (**C**) Elesclomol. (**D**) Gefitinib. (**E**) Lenalidomide. (**F**) Nilotinib. (**G**) Parthenolide. (**H**) Pazopanib. (**I**) Rapamycin. (**J**) Shikonin. (**K**) Sunitinib. (**L**) Temsirolimus. (**M**) Vinblastine. (**N**) Vorinostat.

In order to investigate the differences in efficacy potential between high-risk and low-risk groups, we used the R package ‘pRRophetic’. Patients with low-risk had lower IC50 values and were more sensitive to anticancer drugs including Bexarotene, Dasatinib, Elesclomol, Gefitinib, Lenalidomide, Midostaurin, Nilotinib, Parthenolide, Pazopanib, Rapamycin, Shikonin, Sunitinib, Temsirolimus, Vinblastine, and Vorinostat (Figure 9). Based on these results, risk model may be an effective predictor of anticancer drug efficacy.

### Validation of genes in terms of protein expression

**Figure 10:**
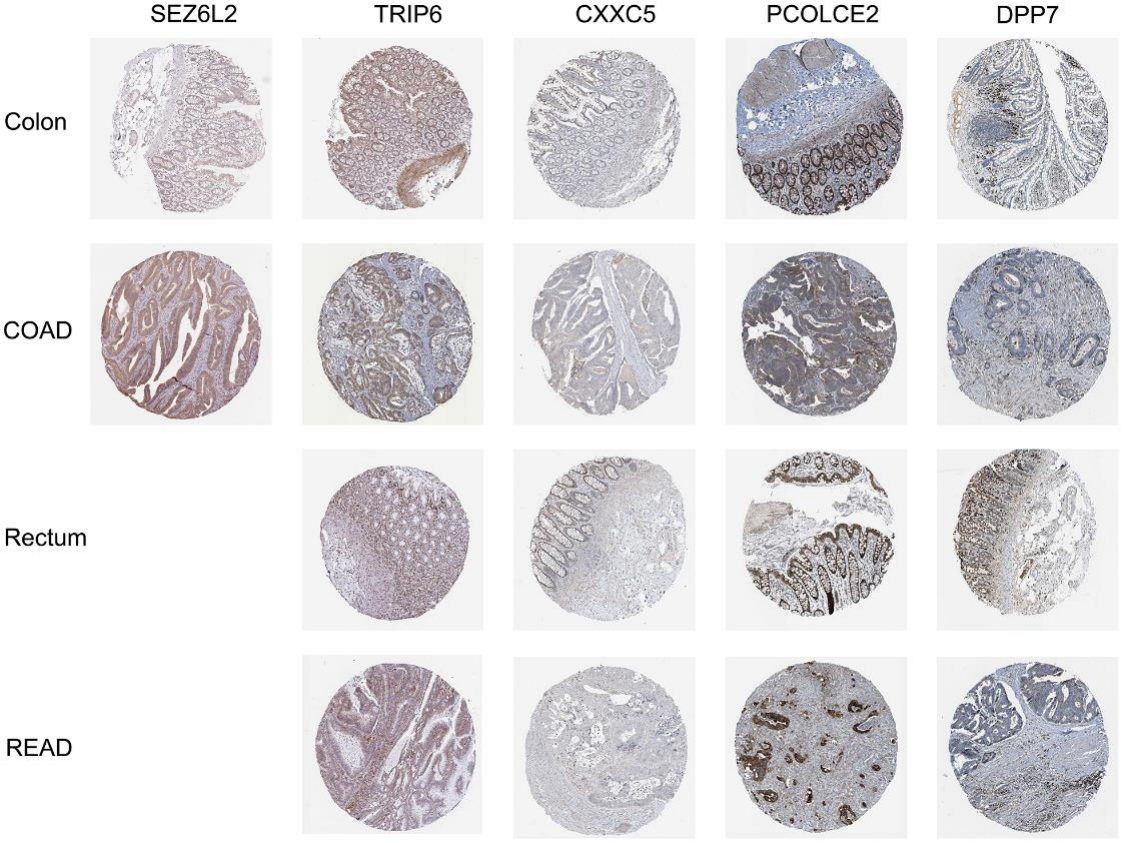
Immunohistochemical stains of the five prognostic genes analyzed from HPA online database.

We found complete protein expression data of 5 genes (TRIP6, CXXC5, PCOLCE2, and DPP7) and the protein expression of SEZ6L2 in the normal colon and CRC tissue in Human Protein Atlas. The IHC staining images were analyzed to compare the protein expression level between normal and tumor tissues (images available from https://v22.proteinatlas.org), the results showed that the expression levels of SEZ6L2, TRIP6, PCOLCE2, and DPP7 in tumor tissues were higher than in normal tissues, while the expression levels of CXXC5 was lower than in normal tissues (Figure 10).

## DISCUSSION

Based on scRNA-seq and bulk RNA-seq data, we identified 8 cell types and 3 significant cell types (Epithelial cells, Fibroblasts and Mast cells) were significantly different between tumor and control samples. In addition, based on marker genes and DEGs, we developed a prognostic model that can be used to stratify CRC patients into high- and low-risk groups in the TCGA and GEO cohorts. The clinical relevance, TIME and drug sensitivity of the various groups were also investigated. As a result of these findings, we are better able to understand the intratumoral heterogeneity in CRC and provide new directions for cancer therapy.

The heterogeneity of fibroblasts played an important role in modulating tumor immune microenvironment [23]. Histologically and biologically, fibroblasts play a key role in CRC initiation, progression, and metastasis [24, 25]. Fibroblasts contribute to carcinogenesis by secreting growth factors, cytokines, and proangiogenic factors [24, 26]. Cancer-associated fibroblasts transfer exosomal lncRNA H19 to promote stemness and chemoresistance [27]. The activation of STAT3 by IL-6/IL-11 in fibroblasts promotes the development of colorectal cancer [28, 29]. As cancer promoters, Mast cells participate in immunosuppression, release proangiogenic and mitogenic factors [30, 31]. Mucosal mast cells activated by inflammation recruit and modulate inflammatory CD11b (+) Gr1(+) cells to promote colon cancer development [32]. The Mast cells activity inhibited CD8+ cell infiltration in CRC tumors, promoting cell engraftment [33]. By secreting Cystatin C, mast cells inhibit colorectal cancer development [34]. Several studies have demonstrated the importance of Fibroblasts and Mast cells in the development of CRC.

The prognostic signature was consisted of 8 marker genes, including ETS2, SEZ6L2, TRIP6, ATOH1, CXXC5, CLDN23, PCOLCE2, and DPP7. It is known that ETS2, a proto-oncogene belonging to the erythroblastosis virus E26 family, is overexpressed in a wide range of human cancers, which include colorectal cancer. It has been shown *in vitro* that the overexpression of ETS2 increases malignant cell behavior (growth, migration, invasion, and resistance to L-OHP) [35]. Seizure-related 6 homolog (mouse)-like 2 (SEZ6L2) belongs to the seizure-related gene 6 family (SEZ6), which is mainly expressed in the brain. In CRC, knockdown of SEZ6L2 promotes the apoptosis of tumour cells, indicating that it might serve as a potential target for therapy [36]. TRIP6 is an adapter protein that binds to thyroid hormone receptors, through its LIM domain, TRIP6 interacts with a wide variety of proteins and act as transcriptional adapters and auxiliary activators [37]. Colorectal cancer cells are regulated by Trip6, a miR-7 target, through regulating proliferation and metastasis [38]. TTPAL activates Wnt by stabilizing TRIP6/ β- Catenin signal promotes colorectal cancer [39]. ATOH1 regulates intestinal progenitors downstream of Notch signaling and activates MUC2 transcription in goblet cells [40, 41]. It was found that SCF/c-KIT signaling promotes the production of MUC2 and Mucinous Colorectal Adenocarcinoma (MCA) tumorigenesis by maintaining the expression of ATOH1 [42]. CXXC5, also known as retinoid-induced nuclear factor, belongs to the CXXC zinc-finger protein family [43]. The role of CXXC5 in colorectal cancer will require further study. CLDN23 encodes a claudin family member, and claudins are known to play a crucial role in cancer growth and progression [44, 45]. Several studies have demonstrated that CLDN23 expression is significantly decreased in CRC tissue, which correlates with a shorter overall survival rate [46]. It has been shown that CLDN23 expression is epigenetically controlled. The PCOLCE2 gene encodes a functional procollagen c-protease enhancer. Activating PCOLCE2 allows type I procollagen to be cleaved to produce mature fibrils [47]. In a previous study, patients with rectal cancer were found to have mutations in PCOLCE2 [47, 48]. It was found that DPP7/2 correlated significantly with poor prognosis in CRC patients [49]. Therefore, these genes play an important role in the prognosis and progression of colorectal cancer.

Based on the 8 signatures, we generated a risk model and divided patient into two groups (high- and low-risk groups). The predictive ability of this model was also confirmed using five external validation cohorts, where consistent results were observed. TME plays an important role in the antitumor response and can influence the prognosis in a significant way [50], we investigated the immune function and relationship between risk groups and 22 immune cells. First, we assessed immune function and drug sensitivity among different risk groups. In high-risk group the Notch signaling pathway is aberrantly activated. The activation of the NOTCH signaling has been proved to promote colorectal cancer invasion and metastasis [51–56] and is associated with poor prognosis. In colorectal cancer, enhanced anti-PD-1 response with microsatellite stability through remodeling Chondroitin-6-Sulfate-Mediated Immune Exclusion [57]. In a model of a highly malignant colorectal tumor, chondroitin sulfate chains are increased to promote epithelial-mesenchymal transition and chemoresistance [58]. The ECM interacts with receptors on the cellular surface and regulates cell behavior, cell proliferation, adhesion, and migration [59, 60]. In a recent study, Nersisyan S et al. revealed that ECM and cellular receptors interact to contribute to the progression and metastasis of colorectal cancer [61, 62]. In low-risk group, terpenoid backbone biosynthesis is activated. CRC occurrence can be influenced by CUBN via its ability to regulate terpenoid backbone biosynthesis [63]. In addition, some studies suggest polymorphisms of the peroxisome proliferators-activated receptor gamma and the risk of colorectal cancer [64]. Pathways' mechanistic role in colorectal cancer deserves further investigation. Next, the high-risk group had a higher proportion of CD8+ T cells resting, NK cells, dendritic cells and Macrophages, indicating this group may be in an active state of antitumor immunity. The high-risk group showed greater immune infiltration and an increased immune response, suggesting they would benefit from immunotherapy.

In order to better guide the treatment of CRC, risk groups of patients were analyzed for drug sensitivity. We investigated 14 anticancer drugs, including Bexarotene, Dasatinib, Elesclomol, Gefitinib, Lenalidomide, Midostaurin, Nilotinib, Parthenolide, Pazopanib, Rapamycin, Shikonin, Sunitinib, Temsirolimus, Vinblastine, and Vorinostat. In the low-risk group, 14 anticancer drugs were found to be sensitive, which provided guidance for deciding which chemotherapy drugs to use. Further investigation of these drugs' clinical significance for CRC patients will be conducted in the follow-up study.

## CONCLUSIONS

In this study, scRNA-seq and bulk RNA-seq data were combined to construct and validate a CRC prognostic model. We identified three significant cell types. Additionally, we identified two risk groups with different prognoses, clinical characteristics, and immune characteristics. High riskscore was associated with poor survival outcomes, high-stage tumors, metastasis, and low sensitivity to chemotherapy. We provide new theoretical insights into the prognosis and precision therapy of CRC patients using scRNA-seq markers.

## Supplementary Material

Supplementary Figures

Supplementary Table 1
