# Supplementary material for: Integrating scRNA-seq and bulk RNA-seq to characterize infiltrating cells in the colorectal cancer tumor microenvironment and construct molecular risk models
Source: Aging (Albany NY). 2023 Dec 5;15(23):13799–821. doi: 10.18632/aging.205263 (PMC10756133; doi:10.18632/aging.205263)
Supplement: Supplementary Figures [file aging-15-205263-s001.pdf]

SUPPLEMENTARY FIGURES

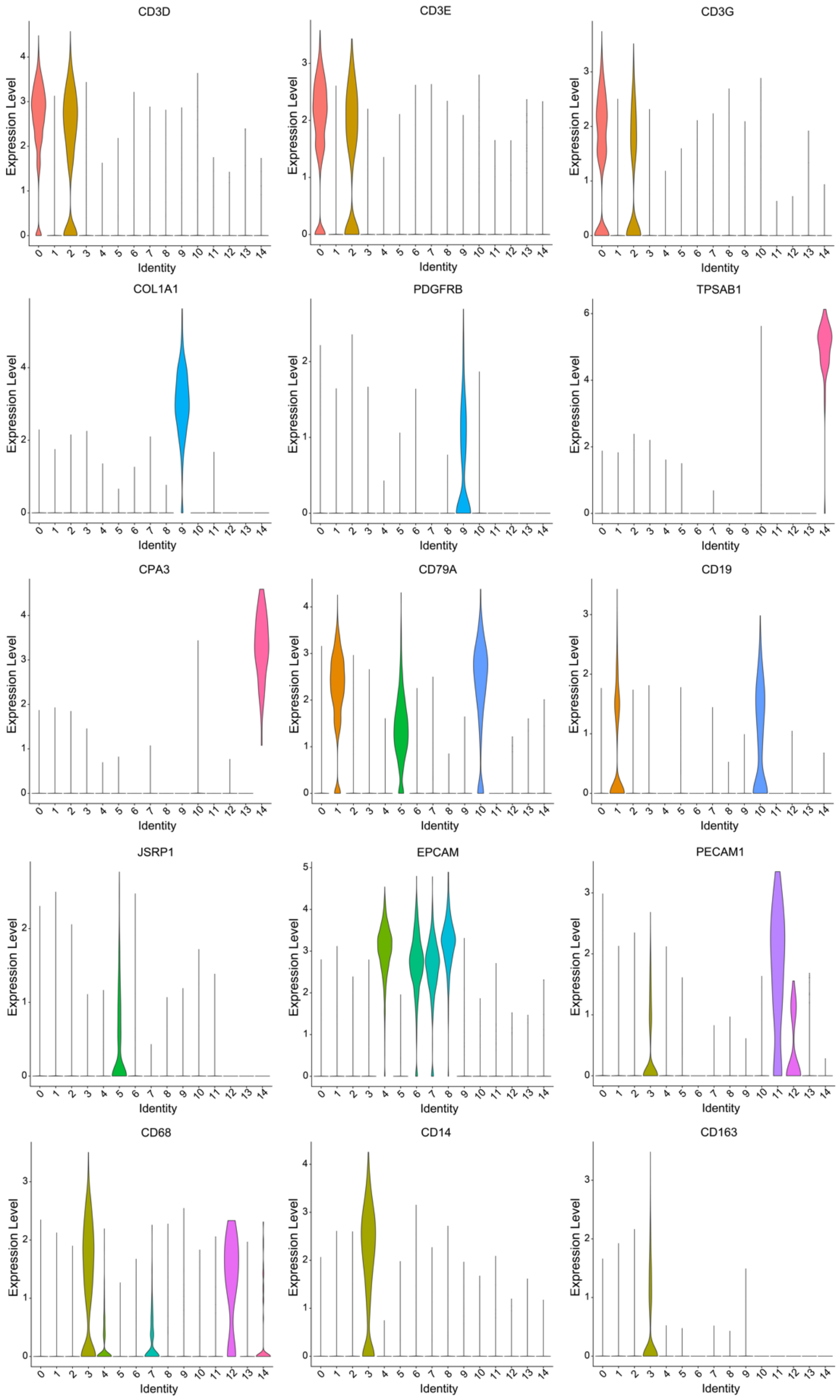

Supplementary Figure 1. Violin diagram displaying the expression of representative markers across the 15 clusters identified in CRC. The y axis shows the normalized read count.

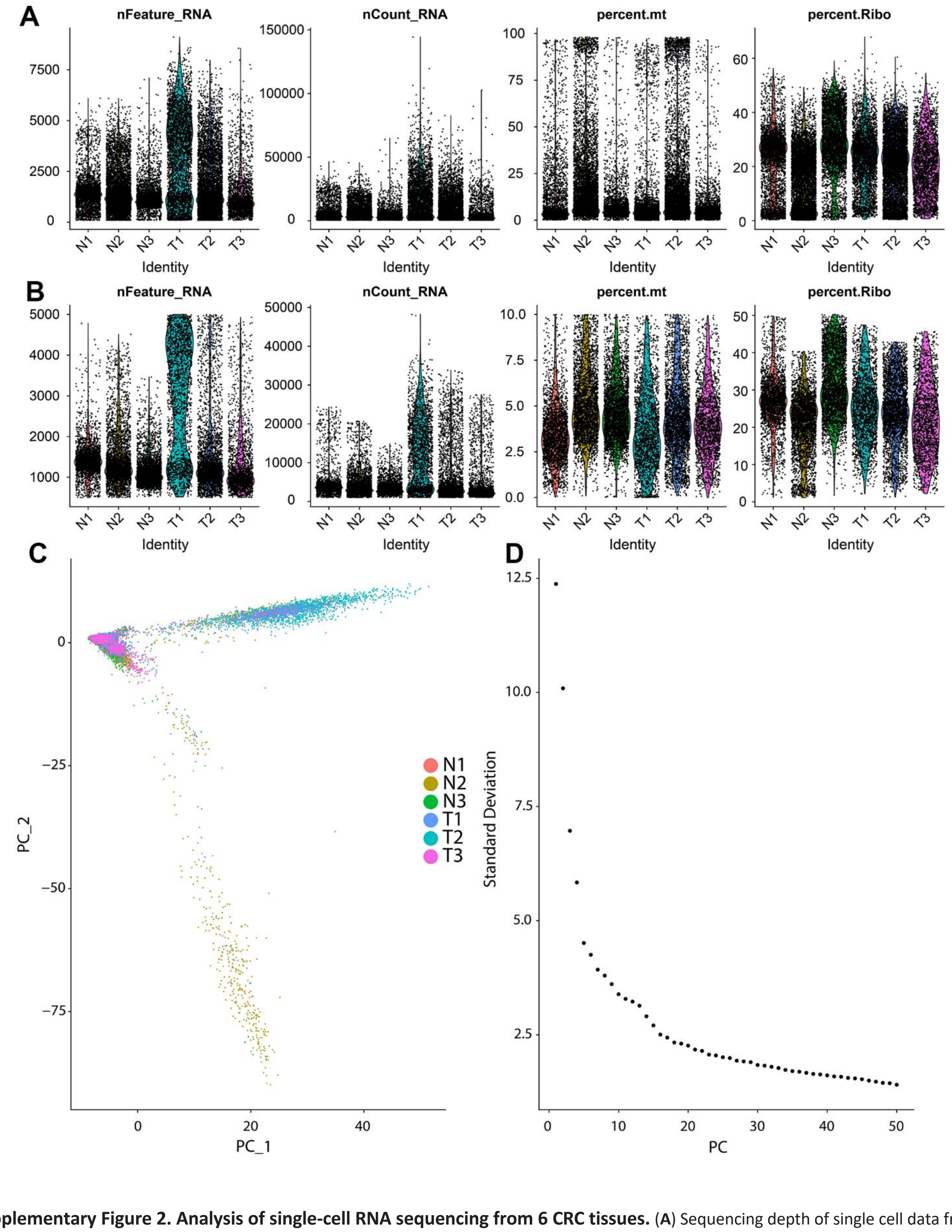

Supplementary Figure 2. Analysis of single-cell RNA sequencing from 6 CRC tissues. (**A**) Sequencing depth of single cell data from 6 patients with colorectal cancer. The plots show the total number of expressed genes (nFeature_RNA), number of transcripts (nCount_RNA), percentage of mitochondrial transcript expression ratio (percent.mt), and Ribosome transcript expression ratio (percent.Ribo) from CRC and HC samples in unfiltered single cell data. (**B**) The plots of filtered single cell data show the total number of expressed genes (nFeature_RNA), number of transcripts (nCount_RNA), percentage of mitochondrial transcript expression ratio (percent.mt), and Ribosome transcript expression ratio (percent.Ribo) from CRC and HC samples. (**C**) PCA plot of single-cell sequencing profiles from 6 samples. (**D**) The standard deviation of 1–5 PCs calculated using ElbowPlot function.

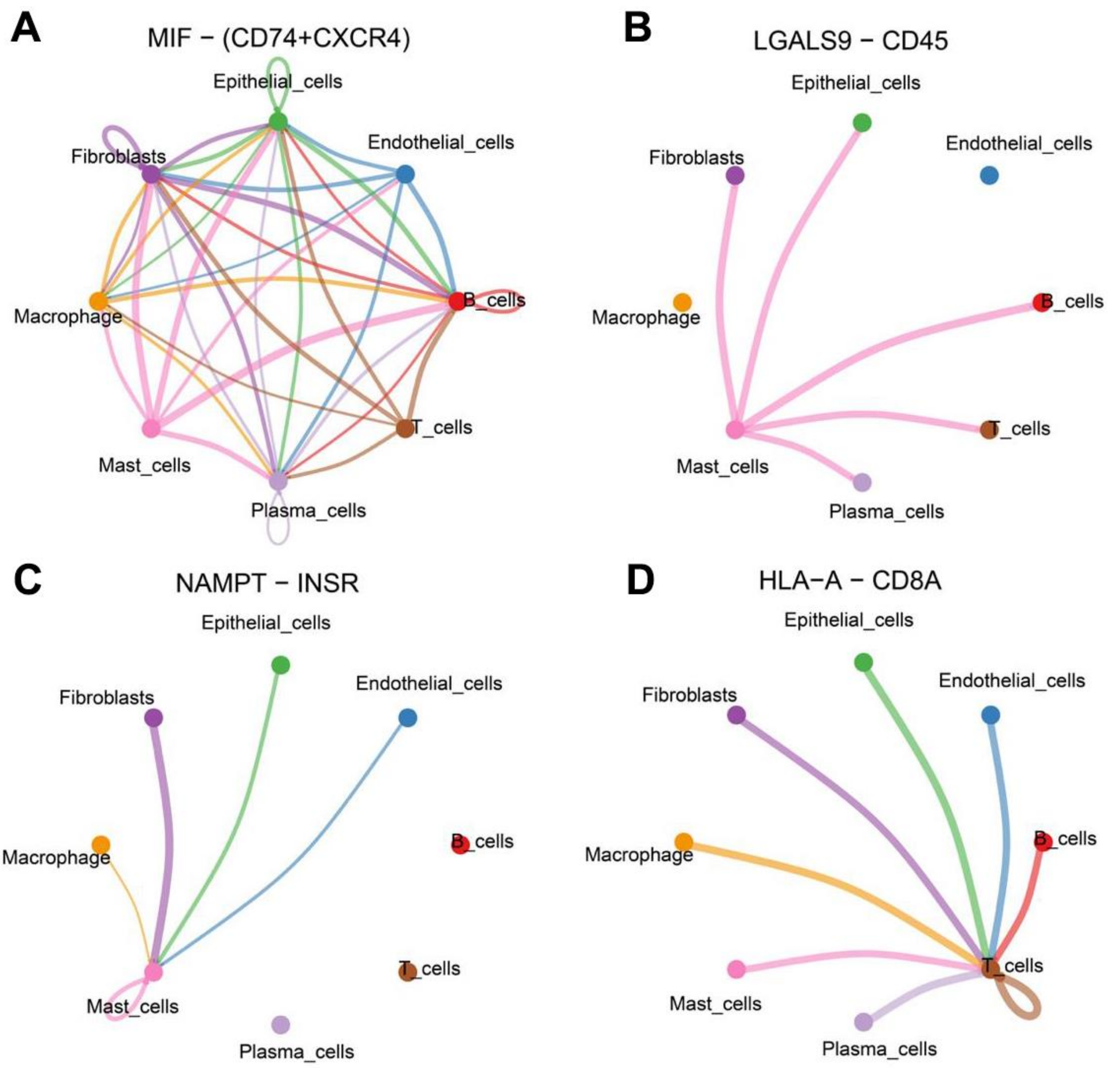

Supplementary Figure 3. Ligand-receptor interaction atlas within four pathways. (**A**) CD74-CXCR4 interaction atlas within MIF signaling pathway. (**B**) LGALS9-CD45 interaction atlas within GALECTIN signaling pathway. (**C**) NAMPT-INSR interaction atlas within VISFATIN signaling pathway. (**D**) HLA-A-CD8A interaction atlas within MHC−I signaling pathway.
